# Validity of Valence Estimation of Dopants in Glasses using XANES Analysis

**DOI:** 10.1038/s41598-017-18847-0

**Published:** 2018-01-11

**Authors:** Hirokazu Masai, Toshiaki Ina, Shun Okumura, Ko Mibu

**Affiliations:** 1 Department of Materials and Chemistry, National Institute of Advanced Industrial Science and Technology, 1-8-31 Midorigaoka, Ikeda, Osaka, 563-8577 Japan; 2 Japan Synchrotron Radiation Research Institute (JASRI/SPring-8), Kouto, Sayo-cho, Hyogo, 679-5198 Japan; 3 Institute for Chemical Research, Kyoto University, Gokasho, Uji, Kyoto, 611-0011 Japan; 4 Department of Physical Science and Engineering, Nagoya Institute of Technology, Gokiso-cho, Showa-ku, Nagoya, 466-8555 Japan

## Abstract

X-ray absorption near edge structure (XANES) measurement is one of the most powerful tools for the evaluation of a cation valence state. XANES measurement is sometimes the only available technique for the evaluation of the valence state of a dopant cation, which often occurs in phosphor materials. The validity of the core excitation process should be examined as a basis for understanding the applicability of this technique. Here, we demonstrate the validity of valence estimation of tin in oxide glasses, using Sn K-edge and L-edge XANES spectra, and compare the results with ^119^Sn Mössbauer analysis. The results of Sn K-edge XANES spectra analysis reveal that this approach cannot evaluate the actual valence state. On the contrary, in L_II_-edge absorption whose transition is 2p_1/2_-d, the change of the white line corresponds to the change of the valence state of tin, which is calculated from the ^119^Sn Mössbauer spectra. Among several analytical approaches, valence evaluation using the peak area, such as the absorption edge energy *E*_0_ at the fractions of the edge step or *E*_0_ at the zero of the second derivative, is better. The observed findings suggest that the valence state of a heavy element in amorphous materials should be discussed using several different definitions with error bars, even though L-edge XANES analyses are used.

## Introduction

Phosphors plays an important role in industrial and medical fields. For conventional crystalline phosphors, an important feature is the ability to control the valence state and the local coordination field of the activator, i.e. the emission centre, which dominates the performance of the material^1–5^. In the case of ordered crystals, even though the crystallites are nano-sized, X-ray and neutron diffraction analyses are the most powerful techniques for the precise establishment of a target structure. Conversely, for amorphous materials, it is necessary to examine the target structure with several measurement techniques because of the lack of an ordered structure. In particular, in amorphous materials, it is extremely difficult to visualize the local coordination state in a small amount of a particular component, such as a dopant in a matrix. In these cases, X-ray absorption fine structure (XAFS) measurements is often one of the techniques used to evaluate the local coordination^6–12^. Both the extended XAFS (EXAFS) regions, which are obtained by complexing the diffracted X-ray, and the X-ray absorption near edge structure (XANES), provide information about the valence state and coordination of the target cation.

In order to obtain valence estimation, XAFS analysis is widely used in synchrotron radiation facilities such as SPring-8 (Hyogo, Japan) or the Photon Factory (Tsukuba, Japan). This method has the following advantages; (1) provides structural information not only of crystals, but also of amorphous substrates (including liquids), (2) provides structural information for trace amount of various elements (ppm order), and (3) facilitates non-destructive measurement of various shapes and sample states, even *in situ*, using the high permeability of X-rays. In particular, non-destructive XAFS method can be used as an effective analysis tool for samples with complex and heterogeneous compositions, including trace amount of elements. This technique is therefore a powerful approach for determining the valence state and the local symmetry of various cations, which is not usually facilitated using other measurement techniques. Examinations of heavier cations is generally performed using L-edge XAFS measurement^8–12^. On the other hand, recent measurement techniques and equipment using the K-edge XAFS analysis have been performed for even heavier cations^13–17^. By using the K-edge XAFS, the EXAFS region can be obtained in a wide *k* range, which is quite different from the L-edge analysis, in which the EXAFS region is restricted by each L-edge. However, the observed change in the absorption edge energy *E*_0_ of the K-edge may be too small relative to the resolution of the measurement to determine the origin, especially in heavier cations in amorphous materials^15–17^. This is due to the ambiguity of the s-p transition in the K-edge absorption, due to the heavy atom effect. Although we can measure both K- and L-edge XAFS spectra, there is no clear metric for determining the difference in the valence estimation between the K-edge and L-edge, especially in glass materials. Considering the accuracy of each XAFS measurement, alternative methods should be considered in the evaluation.

In this report, we focus on valence estimation via conventional XANES analysis using a Sn target element. We selected Sn because the valence state can be also evaluated using ^119^Sn Mössbauer spectroscopy^16,18–21^. Although Mössbauer spectroscopy is a powerful analysis method for estimating the coordination state using isomer shift, the necessary radiation sources for ^119^Sn Mössbauer spectroscopy are not readily available. Recently, our group has demonstrated the photoluminescence of RE-free glass phosphors containing the Sn^2+^centre, which is an ns^2^-type emission centre that exhibits parity-allowed excitation (^1^ *S*_0_ → ^1^ *P*_1_)^16,17,22–31^. The Sn^2+^centre in oxide glasses exhibit a high UV-excited emission, comparable to that of crystal phosphors such as MgWO_4_^16,17,26–31^. However, in Sr-containing materials, since the absorption of ^119^Sn Mössbauer γ-rays of the Sr cation is larger than that of the lighter cations, it is difficult to estimate the Sn^2+^/Sn^4+^ ratio in Sr-containing materials using ^119^Sn Mössbauer spectroscopy^17^. Since the Sr cation is often used as a key component in various phosphors^32,33^, the ability to examine their valence states via an alternate approach such as XANES, is potentially important.

In this study, we measured the valence state of Sn using both K- and L-edge XAFS analyses, as well as ^119^Sn Mössbauer analysis. Our aim was to examine the relationship between the valence state of tin calculated using ^119^Sn Mössbauer spectroscopy, Sn K- and L-edge XANES analyses. Glasses with different Sn^2+^/Sn^4+^ ratios were prepared by tuning both the preparation atmospheres, viz. Ar and air, and the starting materials, viz. SnO and SnO_2_. Based on previous reports on Sn-doped oxide glasses^16,30^, two kinds of base glasses were selected: 1SnO_α_–60ZnO–40P_2_O_5_ and 1SnO_β_–60ZnO–40B_2_O_3_ (in molar ratio), denoted by SZP and SZB, respectively.

**Figure 1:**
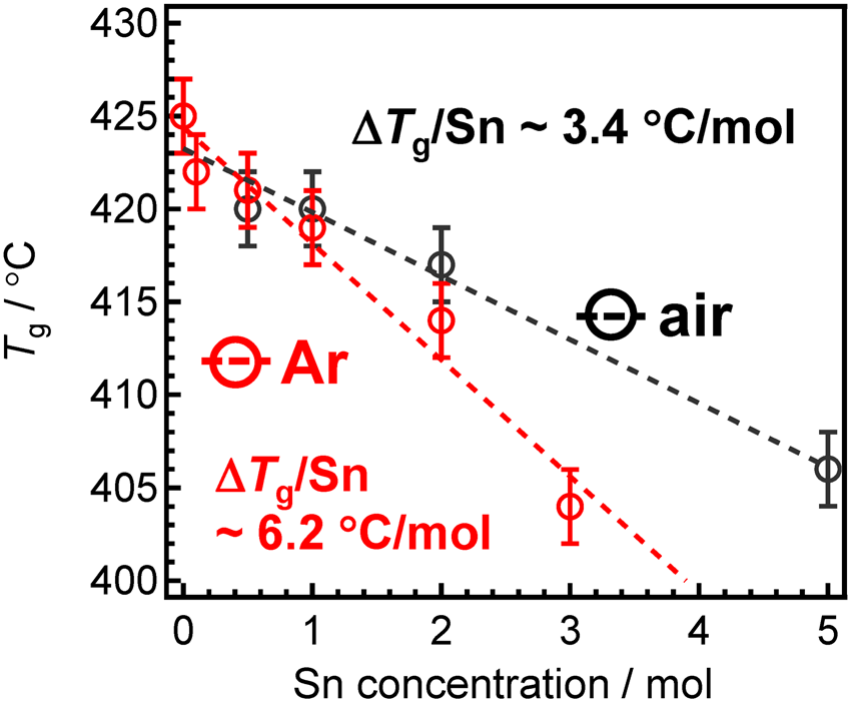
*T*_g_ values for tin-doped ZnO-P_2_O_5_ glasses. *T*_g_ values for the Sn-doped 60ZnO-40P_2_O_5_ (SZP) glasses as a function of the Sn amount. The slope of the SZP glasses prepared in the Ar atmosphere is larger than that of the glass prepared in air.

**Figure 2:**
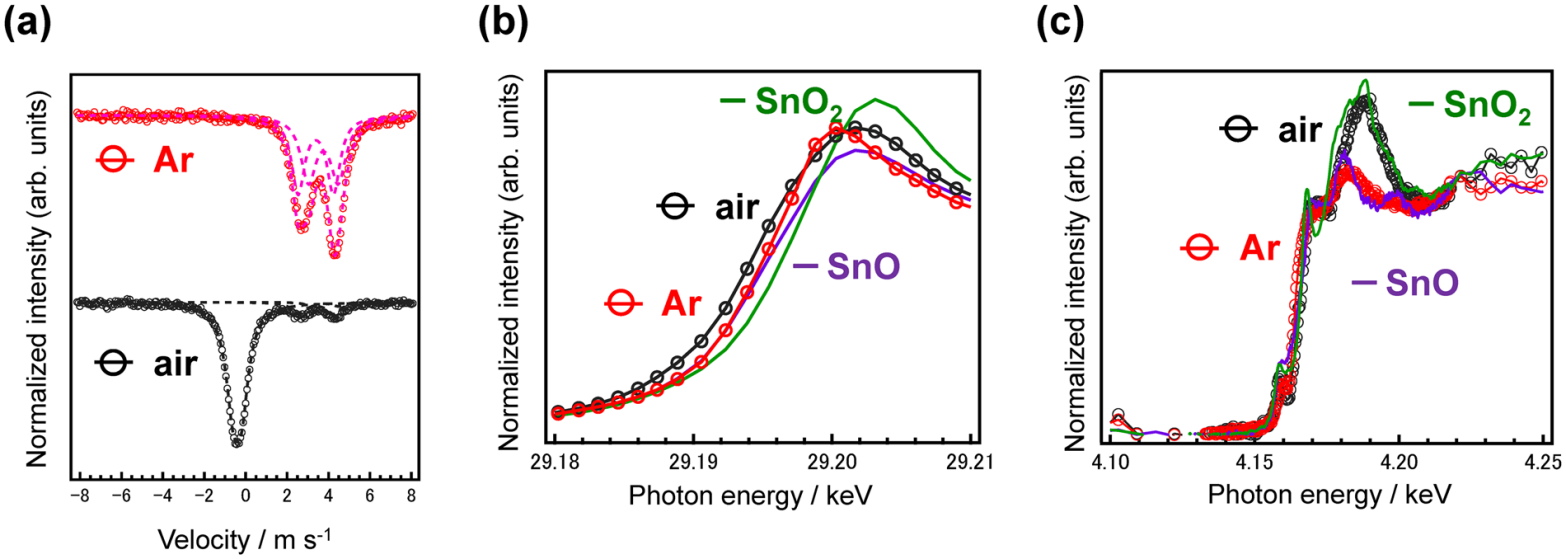
Valence state of tin in the ZnO-P_2_O_5_ glasses. (**a**) ^119^Sn Mössbauer spectra of the SZP glasses. XANES spectra of Sn K-edge (**b**) and Sn L_II_-edge (**c**), respectively. The XANES spectra of SnO and SnO_2_ are also shown for reference. The dashed lines in (**a**) are the fitting lines for two Sn^2+^ and a Sn^4+^ species.

The SZP and SZB glasses were colourless, transparent and independent of the melting atmosphere. Figure 1 shows the glass transition temperature *T*_g_ of the SZP glasses with different Sn concentrations, melted in Ar and air. Although *T*_g_ decreases with an increase in the amount of Sn in both cases, the rate at which they decrease varies. The Sn-doped ZnO–P_2_O_5_ glasses melted in Ar, produced a steeper slope compared with glasses melted in air. It has been reported that the Sn^2+^ species induce a larger decrease in *T*_g_ than the Sn^4+^ species^34,35^. In other words, the value of *T*_g_ may reflect the valence state of Sn, and the greater the Sn^4+^ ratio, the greater the increase in *T*_g_. Since this difference suggests the oxidation of the SnO species, we can conclude that several Sn^2+^ species are oxidized during air melting. Figure 2a shows the ^119^Sn Mössbauer spectra at room temperature for the SZP glasses melted in Ar and air atmospheres. The starting material for tin was Sn(II)O. The peak at 2–4 mm s^−1^ corresponds to the Sn^2+^ species, whereas the peak at 0 mm s^−1^ corresponds to the Sn^4+^ species^18–20^. This figure indicates that most of the Sn in the glass melted in air, exists as Sn^4+^, whereas Sn^4+^ was not observed in the glass melted in the Ar atmosphere. After peak deconvolution, the amounts of Sn^2+^ in the SZP glass melted in air and in Ar were calculated as 14% and ~100%, respectively, ignoring the difference in the recoilless fraction between the Sn^4+^ and Sn^2+^ sites. This suggests that some of the Sn^2+^ species were oxidized into Sn^4+^ during air melting, which is also indicated by the change in *T*_g_ values.

Figure 2b shows the Sn K-edge XANES spectra of the SZP glasses melted in the Ar and air atmospheres. The spectra of SnO and SnO_2_ are also shown for comparison. Since a higher absorption edge indicates a higher oxidation state of the cation, we take the absorption edge energy *E*_0_, to be the energy at the zero-crossing of the 2nd derivative. The *E*_0_ of the SZP glass prepared in air is lower than that of the glass melted in Ar. This suggests that the amount of Sn^2+^ in the former is higher than that of the latter. In order to check for any inconsistency between the Sn^2+^ ratio estimated from *E*_0_ and that calculated from the Mössbauer spectra, we prepared several glass samples that were melted in Ar and air.

Supplementary Figure 1 shows the Sn K-edge XANES spectra of SZP glasses with different Sn concentrations, melted in Ar and air, with Sn-foil, SnO, and SnO_2_ also shown for reference. Supplementary Table 1 lists the Δ*E*_0_ values obtained by subtracting the *E*_0_ value of the Sn-foil (Suppl. Figure 1) from the SZP glasses containing different amounts of Sn. From these data, it is observed that the *E*_0_ of the SZP glass prepared in air is lower than that of SnO. This indicates that there is a difference between the real valence state of Sn obtained from the ^119^Sn Mössbauer spectra, and the evaluated valence state of Sn from the K-edge XANES spectra. Since the measurement resolution is ΔE/E ~6 × 10^−5^, a difference of less than 1.75 eV is insignificant. Therefore, a quantitative analysis of the Sn^2+^ ratio from the *E*_0_ of K-edge XANES spectra will be difficult. However, the peak height of the steep peak near an absorption edge, also-called the ‘white line’, is sometimes used for the evaluation of a valence state^35,36^. If we use the white line height located at approximately 29.2 keV, the peak height of the SZP glass prepared in Ar is comparable to that prepared in air, which suggests that the valence estimation using the K-edge peak height of the white line will also be difficult. Based on the analysis of the ^119^Sn Mössbauer spectra (Fig. 2a), we can conclude that it is difficult to determine the valence state of tin using the *E*_0_ values or the peak height of the white line calculated from K-edge XANES analysis.

Figure 2c shows the Sn L_II_-edge XANES spectra of the SZP glasses, along with the spectra for SnO and SnO_2_. The spectrum of the SZP glass prepared in Ar is similar to that of SnO, while the spectrum of the glass prepared in air is similar to that of SnO_2_, indicating that each preparation atmosphere has a clear effect on the L_II_-edge XANES spectra. It is notable that the peak heights of these glasses are lower than those of the references. Since the observed white line is affected by the coordination symmetry, Sn^2+^ in the SZP glass has a more disordered structure compared with the SnO crystal. This suggests that the valence state of tin can be evaluated from the L_II_-edge XANES spectra, which is indicative of the 2p_1/2_-d energy transition. In contrast to the K-edge XANES spectra whose s-p transition path becomes more obscure with an increase in the atomic number, the observed difference originates from the local coordination state. Considering the fraction of Sn cations compared to the total cation count (1/141), we assume that there is no structural interaction between the Sn cations, and that they are homogeneously dispersed as isolated cations in the zinc phosphate network. Therefore, we conclude that L_II_-edge XANES spectra are suitable for the evaluation of the valence state of tin (dopant) in amorphous glasses.

**Figure 3:**
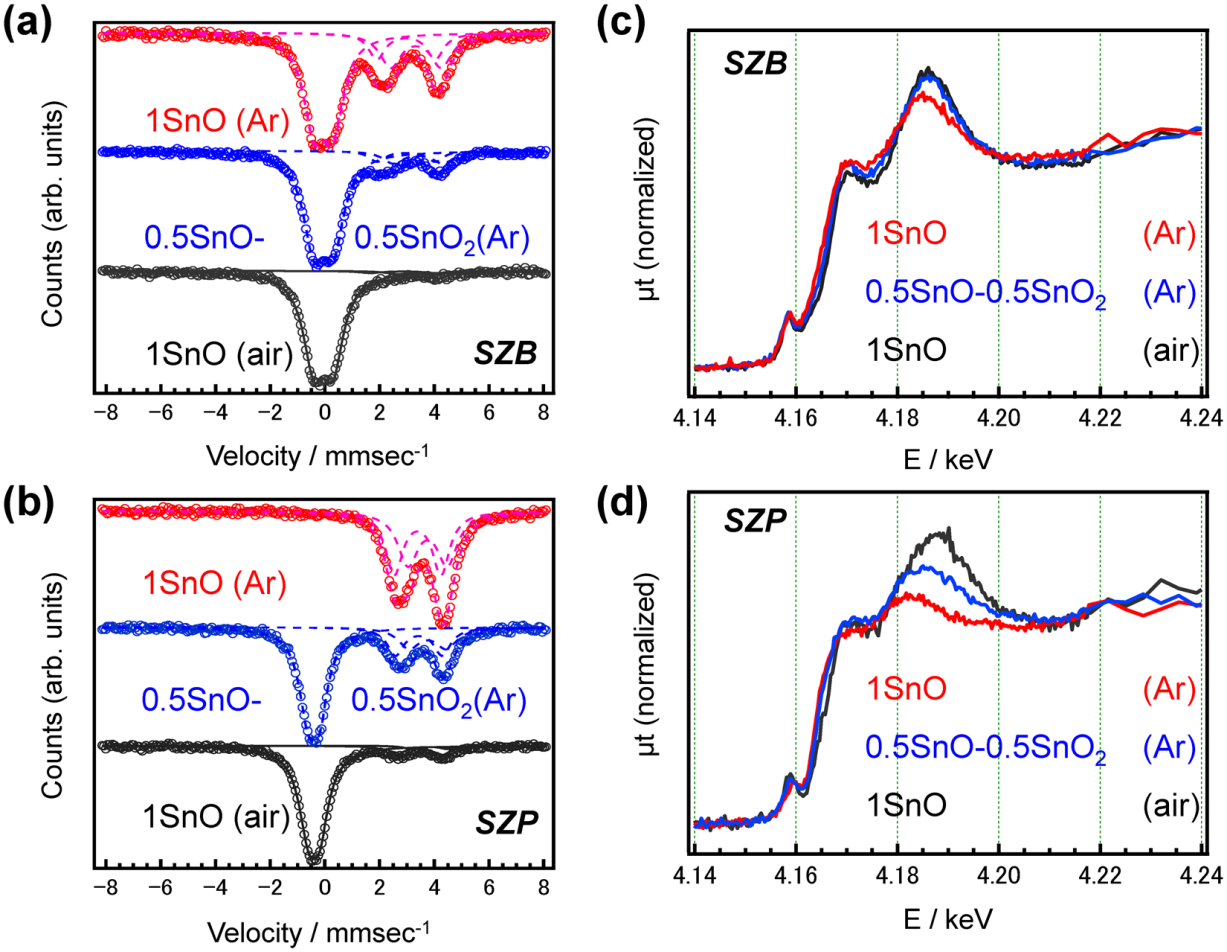
Comparison of the ^119^Sn Mössbauer and Sn L_II_-edge XANES spectra. The ^119^Sn Mössbauer spectra of SZB (**a**) and SZP (**b**) glasses. Sn L_II_-edge XANES spectra of SZB (**c**) and SZP (**d**) glasses. The figure legends indicate the starting chemicals of the Sn species in each glass and atmosphere. The dashed lines in (**a**) and (**b**) are the fitting lines for two Sn^2+^ and a Sn^4+^ species.

We then prepared different SZP and SZB glasses using the starting material SnO_2_ in Ar and air atmospheres, in order to tailor different Sn^2+^/Sn^4+^ ratios. Figure 3a and b show the ^119^Sn Mössbauer spectra of the SZB and SZP glasses prepared under different conditions. The isomer shifts of Sn^2+^ and Sn^4+^ in SZB glasses are different from those in SZP glasses, which is due to difference in the local coordination states. These Mössbauer spectra confirm that the valence state of tin in the SZP glass is different from that in the SZB glass, even though the preparation conditions are the same. The Sn L_II_-edge XANES spectra of each composition are shown in Fig. 3c and d. The white line intensity of the SZP glass prepared in Ar, whose Sn^2+^ ratio is almost 100%, is the lowest among these glasses. Conversely, the white line intensity of the SZB glass prepared in air is the highest. In addition, the peak energy of the white line shifts to the higher-energy side upon air melting, which corresponds to an energy shift in the absorption edge due to oxidation. Therefore, the white line intensities are correlated with the Sn^4+^ ratios calculated from Fig. 3a and b.

**Figure 4:**
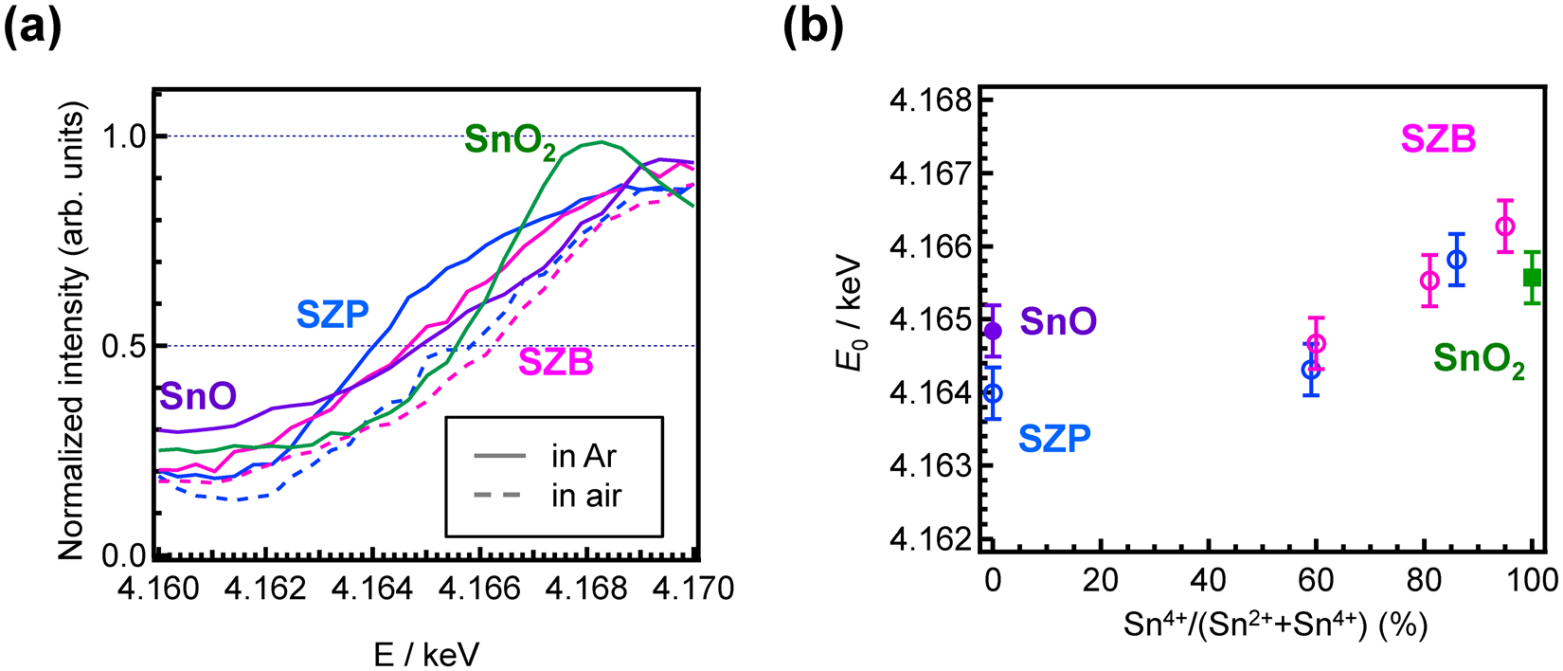
Changes in Sn L_II_ edge XANES spectra depending on the Sn^4+^ concentration I. (**a**) Sn L_II_-edge XANES spectra of the SZB and SZP glasses prepared in Ar (solid lines) and in air (dashed lines). The spectra of SnO and SnO_2_ are also depicted as references. (**b**) The relationship between *E*_0_, which are defined as fractions of the edge step, and the Sn^4+^/(Sn^2+^+Sn^4+^) ratio of the glasses.

Figure 4a shows the Sn L_II_-edge XANES spectra of the SZB and SZP glasses prepared in Ar (solid lines) and in air (dashed lines). The spectra of SnO and SnO_2_ are also depicted as references. For evaluation of the valence state of cations using the XANES technique, several definitions are conventionally used: *E*_0_ energy at the fractions of the edge step, *E*_0_ energy at the zero of the second derivative, and the peak area of a species. Figure 4b shows the relationship between *E*_0_, which is defined as a fraction of the edge step, and the Sn^4+^/(Sn^2+^+Sn^4+^) ratio of the glasses. Although the ^119^Sn Mössbauer spectra suggest the existence of Sn^2+^ and/or Sn^4+^, a ZP glass exhibits a lower *E*_0_ energy than SnO, whereas a ZB glass exhibits a higher *E*_0_ energy than SnO_2_. This clearly indicates that a simple signal convolution of SnO and SnO_2_ is unadoptable for evaluation of Sn cation in glass materials. Such a difference is observed in the *E*_0_ energy at the zero of the second derivative of each sample. Supplementary Figure 2 shows the relationship between *E*_0_, which are defined as the zero of the second derivative, and the Sn^4+^/(Sn^2+^+Sn^4+^) ratio of the glasses. The deviation from the linearity between the valence state and *E*_0_ energy is observed to increase. The valence estimation using the zero of the second derivative, is therefore worse compared to that using the *E*_0_ energy at the fractions of the edge step.

**Figure 5:**
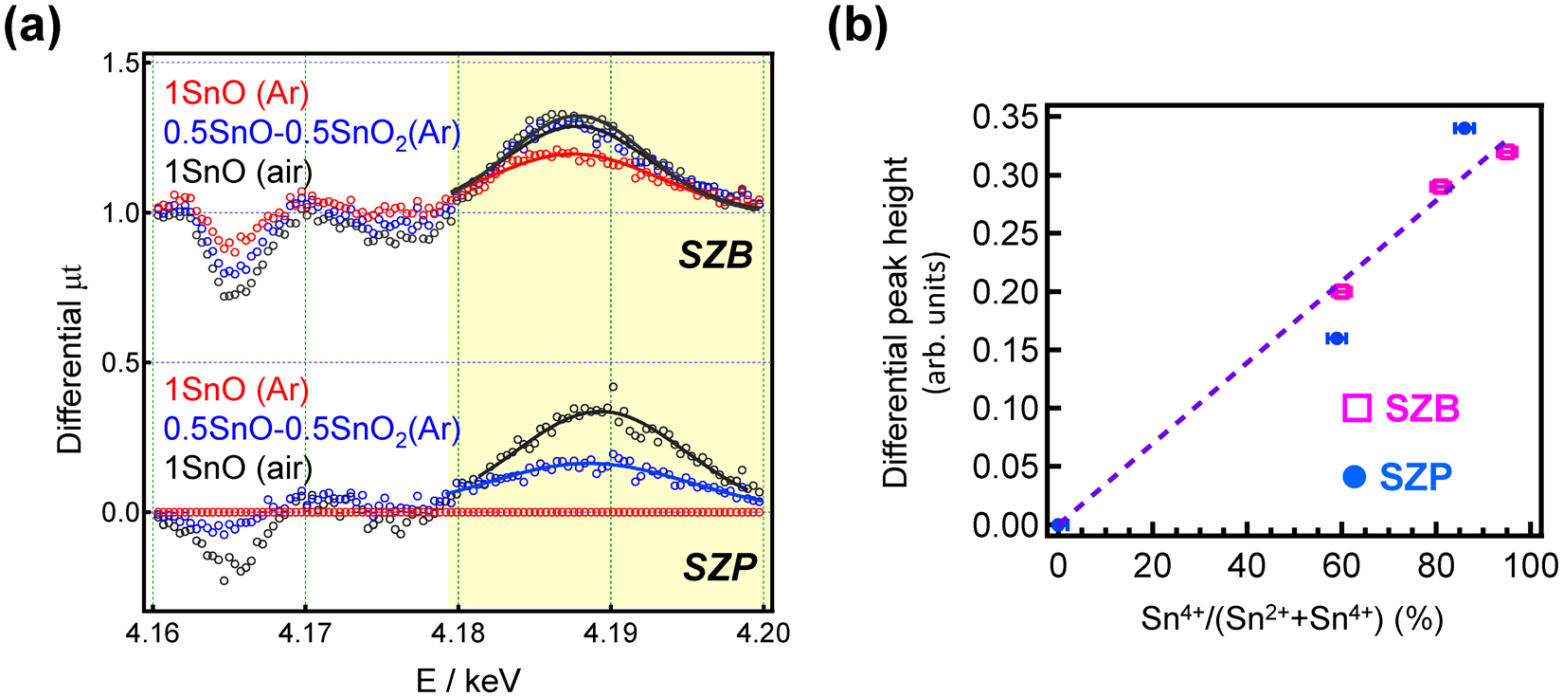
Changes in the Sn L_II_-edge XANES spectra depending on the Sn^4+^ concentration II. (**a**) Differential Sn L_II_-edge XANES spectra of the SZB and SZP glasses, using the SZP glass prepared in Ar as a standard. (**b**) The relationship between differential peak heights at the white line peak (around 4.19 keV) and the Sn^4+^/(Sn^2+^+Sn^4+^) ratio of the glasses.

Considering the aforementioned results, we use the peak area for evaluation of valence state of tin. As previously indicated, the Sn^2+^ ratios in the SZP glasses are almost 100%, which was confirmed by ^119^Sn Mössbauer spectroscopy (see Fig. 3d). Using the SZP glass melted in Ar as a standard, the spectral changes from the Sn^2+^ oxidation can be observed. Figure 5a shows the differential L_II_-edge Sn XANES spectra of the SZP and SZB glasses, which were prepared under different conditions. These spectra were obtained after subtraction of the normalized XAFS spectra of the SZP glass prepared in Ar.

Supplementary Figure 3 shows the L_II_-edge Sn XANES spectra of the SZP glasses and the differential spectra. Although these peak energies are different because of the local coordination state of the Sn cation, the differential peak height can be fitted with a Gaussian function. Figure 5b shows the relationship between the differential peak height at the white line peak, and the Sn^4+^/(Sn^2+^+Sn^4+^) ratio in the SZP and SZB glasses. A linear relationship that appears to be independent of the glass composition was observed. Considering the precision of the ^119^Sn Mössbauer analysis (±2%), we can conclude that these spectra are adequate for the evaluation of the Sn^2+^/Sn^4+^ ratio. Supplementary Figure 4 shows the relationship between the peak height of the pre-edge region (~4.165 keV) and the Sn^4+^/(Sn^2+^+Sn^4+^) ratio of the Sn-doped glasses. In this region, the relationship is non-linear, although they are correlated, and we can conclude that the Sn L_II_-edge measurement is effective in evaluating the valence state of tin.

It has been reported that the L-edge XANES of heavy elements is useful in quantifying their valence states^8–12^, and we have also demonstrated valence estimation using L_III_-edge XANES^37^. However, as previously indicated, there is no available report on the difference in valence estimation between K-edge and L-edge analyses in glass materials. Since it is expected that this difference will be affected by the glass system, *i.e*. the local coordination state of the cation and electrons, we emphasize that the present approach will contribute to a deeper understanding of the local coordination of useful activators in materials science.

In summary, we have examined the correlation between the valence state of Sn in oxide glasses using ^119^Sn Mössbauer spectra as well as Sn K-edge and L_II_-edge XANES analyses. We found that it is difficult to evaluate the valence state of Sn using the K-edge XANES analysis because of an obscure s-p transition. In addition, it is also difficult to determine the valence state from the *E*_0_ value in the L_II_-edge XANES analysis. Conversely, it was determined that the peak height of the white line in L_II_-edge XANES is an indicator for the local coordination state, which is confirmed by the ^119^Sn Mössbauer spectra. Although Sn^2+^ exists in SZP glasses, the white line is broadened compared with the standard SnO, suggesting that the coordination state of Sn^2+^ is not equal, but similar to that of Sn^2+^ in SnO crystals. The peak energy of the white line shift also depends on the actual Sn^2+^ ratio in the glasses, whereas the peak height is independent of the chemical composition of the host glass. Here, we have demonstrated that a valence estimation strongly depends on the estimation approach, even for L-edge analysis. Most industrial glass plates made using the “float method” contain tin at the surface, and the valence states affect the physical properties of the industrial products. The valence state of tin in transparent conducting films is also of significance. Therefore, the present findings are noteworthy, particularly for materials containing tin as a key element. Although the present data are only concerned with tin, we wish to emphasize that our findings are adaptable to other heavy metal cation-doped materials^15^, which is important for a deeper understanding of materials science.

## Experimental Section

### Sample Preparation

The Sn-doped 60ZnO-40P_2_O_5_ (SZP) and 60ZnO-40B_2_O_3_ (SZB) glasses were prepared according to a conventional melt-quenching method by employing a platinum crucible^38,39^. The mixture of ZnO and (NH_4_)_2_ HPO_4_ was initially calcined at 800 °C for 3 h using a Pt crucible in the ambient atmosphere. After treatment, the calcined matrix was mixed with SnO and/or SnO_2_ and melted in an electric furnace at 1100 °C for 30 min in ambient or Ar (5 N) atmosphere. In the case of inert melting, the mixture was set in the atmosphere-controlled electric furnace at room temperature. It took 2 h to heat up from r.t. to 1100 °C, and the temperature was fixed at this value for 30 min. Before initiating the heating, an Ar purge process was performed in the furnace tube. The air in the tube was removed using a vacuum pump, and subsequently purged using 5 N Ar gas. This purging process was performed three times. The glass melt was quenched on a stainless-steel plate at 200 °C and then annealed at *T*_g_, which was measured by a differential thermal analysis (DTA) for 1 h.

### Characterization

*T*_g_ was determined using a DTA system operating at a heating rate of 10 °C/min, using a TG8120 (Rigaku, Japan). The ^119^Sn Mössbauer spectra, i.e. the absorption spectra of the γ-rays by the ^119^Sn nuclei in the samples, were measured using conventional transmission geometry using a Ca^119*m*^ SnO_3_ source at room temperature. The energy of the γ-rays from the source were modulated by the Doppler effect using a velocity transducer with a constant acceleration mode, and the abscissae of the spectra were identified with the units of the Doppler velocity, as in the literature^20^. The valence states of the Sn atoms, which were detected as the peak positions in the ^119^Sn Mössbauer spectra^20^, were deduced by fitting the measured spectra using the commercial software Normos (made by R. A. Brand, commercially available from WissEl GmbH).

The Sn K-edge (29.3 keV) and L_II_-edge (4.17 keV) of the XAFS spectra were measured at the BL01B1 beamline of the SPring-8 (Hyogo, Japan). The storage ring energy was operated at 8 GeV, with a typical current of 100 mA. The Sn K-edge XAFS measurements were carried out using a Si (311) double-crystal monochromator in the transmission mode (Quick Scan method). Conversely, the Sn L_II_-edge XAFS measurements were carried out using a Si (111) double-crystal monochromator in the fluorescence mode using 19-SSD at r.t. The XAFS data of Sn-foil, SnO, and SnO_2_ were also collected under the same conditions.

## Electronic supplementary material

Supplementary Data
