# Supplementary material for: Validity of Valence Estimation of Dopants in Glasses using XANES Analysis
Source: Sci Rep. 2018 Jan 11;8:415. doi: 10.1038/s41598-017-18847-0 (PMC5764964; doi:10.1038/s41598-017-18847-0)
Supplement: Supplementary file 1 — Supplementary Data [file 41598_2017_18847_MOESM1_ESM.doc]

**Validity of Valence Estimation of Dopants in Glasses using XANES Analysis**

by H. Masai, T. Ina, S. Okumura, and K. Mibu

**Supplementary Data**

**Supplementary Figure 1 |** Sn K-edge XANES spectra of Sn-doped 60ZnO-40P_2_O_5_ (SZP) glasses along with those of references: Sn foil, SnO, and SnO_2_. Although the oxidation reaction might occur in SZP glasses prepared in air, the *E*_0_ energies suggest that there is no difference between glasses prepared in air and Ar atmosphere.

**Supplementary Table 1 |** The ΔE_0_ values of Sn-doped 60ZnO-40P_2_O_5_ (SZP) glasses along with those of references: Sn foil, SnO, and SnO_2_. It is expected that these differences calculated from K-edge XANES analysis have little meaning, because the large amount of Sn^4+^ was observed in ^119^Sn Mössbauer spectroscopy (see Figs. 2 and 3).

|  | **Δ*E*_0_ from Sn foil / keV** | |
| --- | --- | --- |
|  | **air** | **Ar** |
| 1SnO | 1.0 | 2.4 |
| 5SnO | 0.3 | 2.1 |
| SnO | 1.8 | |
| SnO_2_ | 4.5 | |

**Supplementary Figure 2 |** The relationship between *E*_0_, which are defined as the zero of the second derivative, and the Sn^4+^ / (Sn^2+^+Sn^4+^) ratio of the glasses.

**Supplementary Figure 3 |** Spectra change of Sn L_II_ edge XANES spectra depending on the Sn^4+^ concentration. | (a) Sn-L_II_ edge XANES spectra of 1SnO*_x_*-60ZnO-40P_2_O_5_ glasses. (b) Differential Sn-L_II_ edge XANES spectra of 1SnO*_x_*-60ZnO-40P_2_O_5_ glasses, whose standard is that of 1SnO-60ZnO-40P_2_O_5_ glass prepared in Ar.

**Supplementary Figure 4 |** Spectra change of Sn L_II_ edge XANES spectra depending on the Sn^4+^ concentration. | (a) Differential Sn-L_II_ edge XANES spectra of 1SnO*_x_*-60ZnO-40B_2_O_3_ and 1SnO*_x_*-60ZnO-40P_2_O_5_ glasses, whose standard is that of 1SnO-60ZnO-40P_2_O_5_ glass prepared in Ar. (b) Relationship between differential peak peak height of the pre-edge region (~4.165 keV) and Sn^4+^/(Sn^2+^+Sn^4+^) ratio of these glasses.
